# Impact of measurement method on interobserver variability of apparent diffusion coefficient of lesions in prostate MRI

**DOI:** 10.1371/journal.pone.0268829

**Published:** 2022-05-23

**Authors:** Hiroaki Takahashi, Kotaro Yoshida, Akira Kawashima, Nam Ju Lee, Adam T. Froemming, Daniel A. Adamo, Ashish Khandelwal, Candice W. Bolan, Matthew T. Heller, Robert P. Hartman, Bohyun Kim, Kenneth A. Philbrick, Rickey E. Carter, Lance A. Mynderse, Mitchell R. Humphreys, Jason C. Cai, Naoki Takahashi

**Affiliations:** 1 Department of Radiology, Mayo Clinic, Rochester, Minnesota, United States of America; 2 Department of Radiology, Kanazawa University School of Medical Science, Ishikawa, Japan; 3 Department of Radiology, Mayo Clinic, Rochester, Arizona, United States of America; 4 Department of Radiology, Mayo Clinic, Rochester, Florida, United States of America; 5 Department of Health Science Research, Mayo Clinic, Rochester, Florida, United States of America; 6 Department of Urology, Mayo Clinic, Rochester, Minnesota, United States of America; 7 Department of Urology, Mayo Clinic, Rochester, Arizona, United States of America; Medical University of Vienna, AUSTRIA

## Abstract

**Purpose:**

To compare the inter-observer variability of apparent diffusion coefficient (ADC) values of prostate lesions measured by 2D-region of interest (ROI) with and without specific measurement instruction.

**Methods:**

Forty lesions in 40 patients who underwent prostate MR followed by targeted prostate biopsy were evaluated. A multi-reader study (10 readers) was performed to assess the agreement of ADC values between 2D-ROI without specific instruction and 2D-ROI with specific instruction to place a 9-pixel size 2D-ROI covering the lowest ADC area. The computer script generated multiple overlapping 9-pixel 2D-ROIs within a 3D-ROI encompassing the entire lesion placed by a single reader. The lowest mean ADC values from each 2D-small-ROI were used as reference values. Inter-observer agreement was assessed using the Bland-Altman plot. Intraclass correlation coefficient (ICC) was assessed between ADC values measured by 10 readers and the computer-calculated reference values.

**Results:**

Ten lesions were benign, 6 were Gleason score 6 prostate carcinoma (PCa), and 24 were clinically significant PCa. The mean±SD ADC reference value by 9-pixel-ROI was 733 ± 186 (10^−6^ mm^2^/s). The 95% limits of agreement of ADC values among readers were better with specific instruction (±112) than those without (±205). ICC between reader-measured ADC values and computer-calculated reference values ranged from 0.736–0.949 with specific instruction and 0.349–0.919 without specific instruction.

**Conclusion:**

Interobserver agreement of ADC values can be improved by indicating a measurement method (use of a specific ROI size covering the lowest ADC area).

## Introduction

Assessment of diffusion-weighted images (DWIs) and apparent diffusion coefficient (ADC) images plays an important role in classifying prostate lesions by the Prostate Imaging Reporting and Data System (PI-RADS) scoring system. The scoring system relies on qualitative visual assessment and subjective determination of markedly hypointense signal on ADC. The DWI score drives the overall assessment score for peripheral zone (PZ) lesions and influences it for transitional zone (TZ) lesions [1–6]. ADC values have been shown to be the most useful image marker of prostate carcinoma (PCa) aggressiveness [7–12]. A meta-analysis showed that the correlation between ADC values and Gleason score (GS) was moderate (- 0.48) in PZ cancer and mild (- 0.22) in TZ cancer [8]. ADC values may assist differentiation between benign and malignant prostate tissue in the PZ using a threshold of 700 to 900 (10^−6^ mm^2^/s) [3,7]. However, quantitative ADC threshold values are not specified in the PI-RADS scoring system since large variability of ADC values exists between magnetic resonance imaging (MRI) scanner models and also given the inconsistency in how a region of interest (ROI) is placed [13–16]. Standardization of ADC measurement should improve inter-observer variability and possibly accuracy of prostate MRI.

Some studies revealed that 0^th^ to 10^th^ percentile ADC values of 3D-whole-lesion-ROI were accurate in diagnosing clinically significant PCa (csPCa: GS ≥7) [9–11]. The major drawback of this method is that it takes a longer time to draw 3D-ROI than 2D-ROI and also requires a histogram calculation, which is not universally available at all reading stations. Therefore, radiologists usually use 2D-ROI to measure ADC values in the clinical setting. However, previous literature reported lower interobserver agreement and lower diagnostic performance of the 2D-ROI method compared to the 3D-ROI method [9,16]. This could be partly explained by the volatility of ADC values measured by 2D-ROI. We assume that the proportion of aggressive tumor components with lower ADC values could change depending on the size of the 2D-ROI.

The purpose of our multireader study was to compare the inter-observer variability of ADC values measured by 2D-ROI with and without the use of a specific ROI size covering the lowest ADC area. The variability of ADC values measured with different scanner models was also evaluated to see how its magnitude differs from that of interobserver variability.

## Materials and methods

Our institutional review board approved this Health Insurance Portability and Accountability Act (HIPAA)-compliant, retrospective study. All patients had previously consented to the use of their medical records for research.

### Patient enrollment

**Fig 1:**
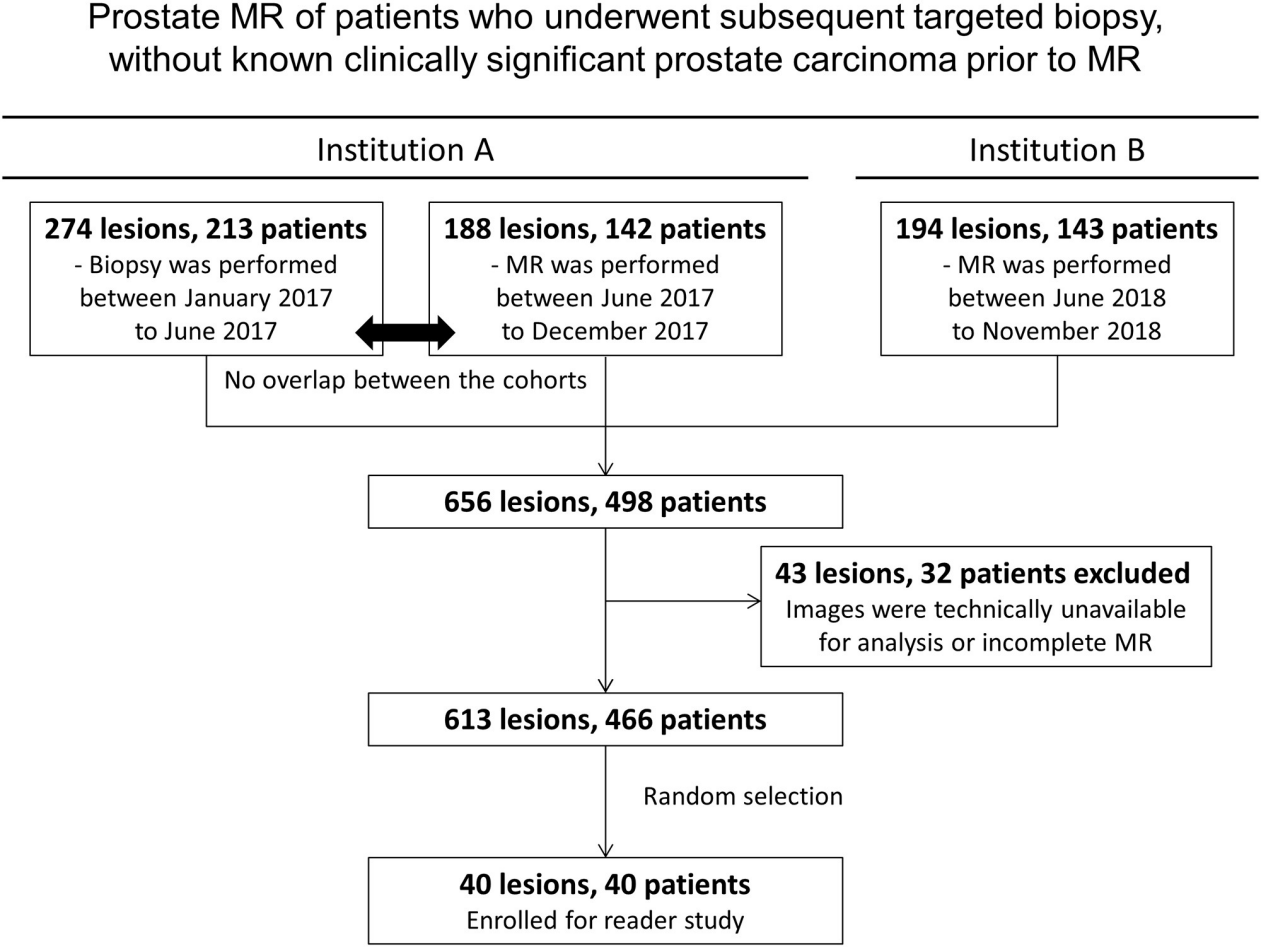
Patient’s enrollment flowchart. At Institution A, 213 patients with a total of 274 lesions underwent biopsy from January 2017, to June 2017, and 142 patients with a total of 188 lesions underwent MRI from June 2017, to December 2017. At Institution B,143 patients with a total of 194 lesions who underwent MRI from June 2018, to November 2018, were enrolled in our study. Thirty-two patients with a total of 43 lesions were excluded because images were unavailable for analysis or MRI was incomplete. A total of 466 patients with a total of 613 lesions were evaluated in this study. Forty patients, each with 1 lesion (40 total lesions) were randomly selected from this cohort for the multireader study.

The details of patient enrollment are shown in Fig 1. Records from a total of 466 patients with 613 lesions amongst them who underwent prostate MRI followed by targeted prostate biopsy without known csPCa clinically significant prostate carcinoma (csPCa: Gleason score [GS] ≥7) prior to MRI) were retrieved. From those, 40 patients, each with 1 lesion (40 total lesions), were randomly selected for a multireader study. The entire cohort was used to compare ADC values taken from different scanner models.

### MRI technique

Among the 40 patients, MRI was performed with a 3.0-T (n = 39) or 1.5-T (n = 1) scanner at our institution (Signa: GE Healthcare or Skyra: Siemens). Transverse fat-suppressed single-shot echo planar DWI was acquired with 2 b values (100 and 800 [n = 32], or 100 and 800 [n = 1]) or 3 b values (100, 400 and 800 [n = 4], or 50, 400 and 800 [n = 3]) (repetition time msec/echo time msec, 3,200–5,200/59-90; section thickness, 4–7 mm; flip angle, 90; FOV, 18–26 cm; pixel spacing, 0.85–1.02 mm). The ADC map was generated using 2 b values or 3 b values. Images were upsampled at the scanner level, and the matrix size was 256 and 256.

## Histopathologic examination

An MRI/US fusion trans-rectal biopsy was performed for the lesions identified on MRI. GS and the location of each lesion were obtained from the official surgical and pathologic reports.

### Multi-reader study

Ten board-certified radiologists (A.K., B.K., N.L., N.T., R.P.H., M.T.H., A.K., A.T.F., C.W.B., and D.A.A. with +20, +20, 20, 20, 18, 14, 11, 10, 10 and 2 years of experience in abdominal radiology) independently measured the ADC values of the lesions using 3 different methods during 3 different sessions. The readers were blinded to the pathologic diagnosis or PI-RADS score. For each case, the lesion was marked with arrows on an axial T2-weighted image to assist reader with lesion detection. The measurement was performed on Visage 7 (Visage Imaging Inc) and/or Invivo DynaCAD (GE Healthcare) (sessions 1 and 2) or Invivo DynaCAD (session 3). Mean ADC value and ROI size were reported in sessions 1 and 2. ADC values of the 10^th^ percentile were reported in session 3.

#### Session 1: Free 2D-small-ROI measurement

Readers measured ADC values by placing a 2D-small-ROI on a single slice as they would usually do in the clinical setting. No other specific instructions were given. Readers were not aware of ROI size used in session 2.

#### Session 2: 2D-small-ROI method with 9 pixels covering lowest ADC area

Readers placed a 2D-small-ROI on a single slice in the area covering the lowest ADC area on Visage 7. ROI size was specified as area (range +/- mm^2^), which corresponds to the 9 pixels.

#### Session 3: 10^th^ percentile of 3D-whole-lesion-ROI measurement

Readers placed a 3D-whole-lesion-ROI encompassing the entire area of visually low on Invivo DynaCAD. The 10^th^ percentile of ADC values was calculated from 3D-whole-lesion-ROI.

### Computed-based calculation for ADC values of lesion

#### 3D-whole-lesion-ROI placement

**Fig 2:**
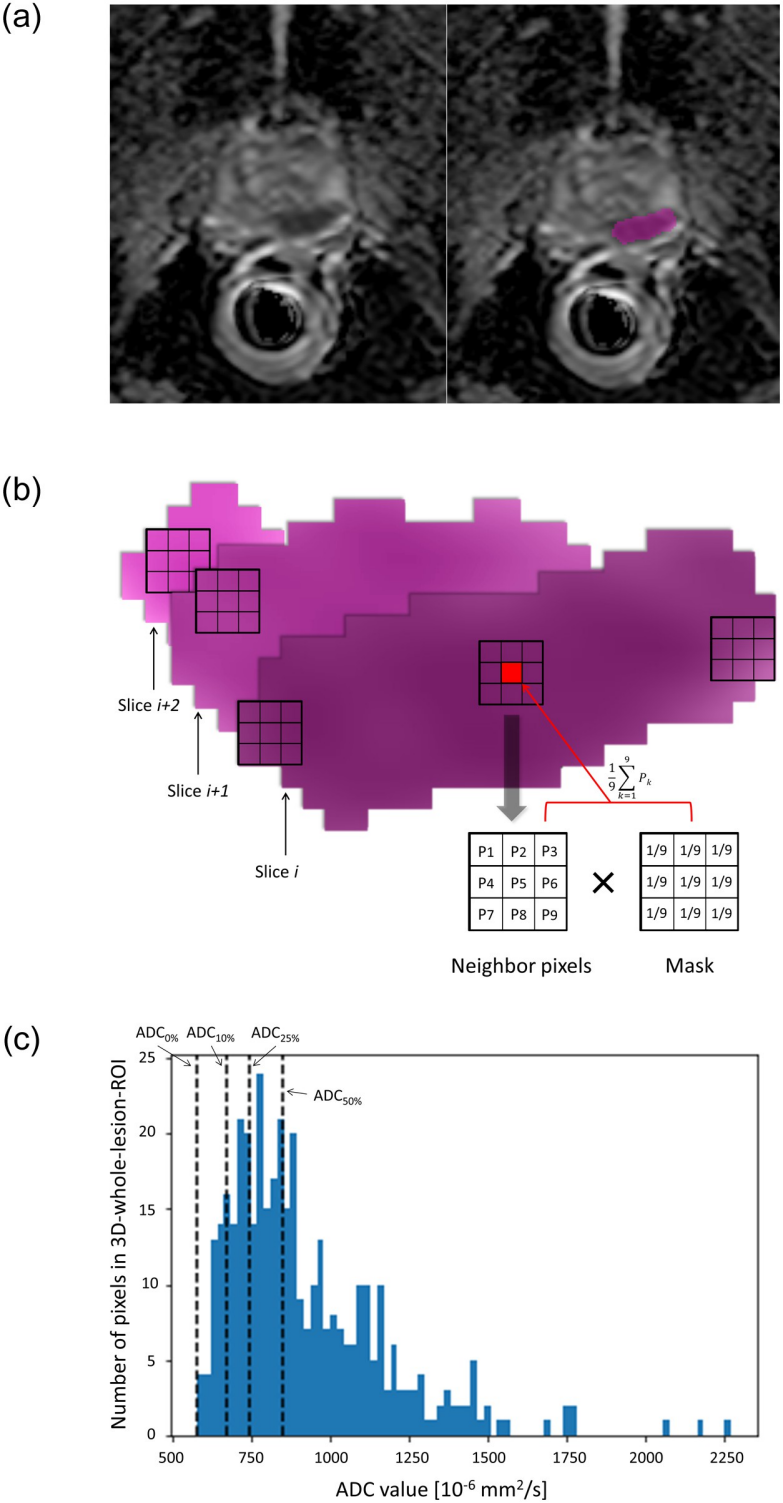
Computed-based calculation for ADC values of lesion. (a) Prostate cancer (Gleason score 4+3) in the left posterior peripheral zone of a 72-year-old man (left). On the ADC image, 3D-whole-lesion-ROI was placed encompassing the entire area of visually low ADC (right). (b) Schematic illustration of multiple overlapping 2D-small-ROIs (9 pixels), which were automatically generated within the 3D-whole-lesion-ROI. The kernel mask was used to average the neighbor pixels to the center pixel. The reference value was determined as the minimum of the multiple mean ADC values of all slices. (c) Histogram of ADC values measured by 3D-whole-lesion-ROI. The 10th percentile ADC values are shown. ADC indicates apparent diffusion coefficient; ROI, region of interest; 2D, two-dimensional; 3D, 3-dimensional.

For each lesion, 3D-whole-lesion-ROI encompassing the entire area of the lesion with visually low ADC values was placed on the ADC images by 1 of 4 board-certified radiologists (A.K., N.L., K.Y. and H.T. with 20+, 20, 11 and 2 years of experience in abdominal radiology) (Fig 2a), using RIL contour (Mayo Clinic) [17]. The radiologists were aware of the location and MRI description of the lesion and were allowed to refer to the sequences other than the ADC images. Two of 4 radiologists also contributed to the multireader study; their reader study was performed after at least 3 months from 3D-whole-lesion-ROI placement to mitigate recall bias.

#### Mean ADC value of lesion by 2D-small-ROI with different pixel sizes

Multiple overlapping 2D-small-ROIs of a certain size were automatically generated within the 3D-whole-lesion-ROI using computer script (Fig 2b). The mean ADC value of pixels of each of the 2D-small-ROIs was calculated. Among the multiple mean ADC values, the lowest mean ADC value was used as a representative value for each ROI size. This process was repeated for 9 different 2D-small-ROI sizes (1, 2, 3, 5, 9, 17, 25, 33, and 49 pixels, square- or near-circular-ROI). If no single 2D-small-ROI of a certain size fit within the 3D-whole-lesion-ROI, the representative value of 1 size smaller ROI was used. The mean ADC value by 2D-small-ROI with 9 pixels was used as reference value for the multi-reader study.

#### 10^th^ percentile ADC value of lesions by 3D-whole-lesion-ROI

A histogram of the ADC values within the 3D-whole-lesion-ROI was created (Fig 2c). ADC values for the 10^th^ percentile were calculated and used as the reference value for the multi-reader study.

### Computed-based calculation for median ADC values of the PZ parenchyma

For all patients (N = 466), the PZ of the prostate parenchyma was segmented using deep-learning assisted manual segmentation. A deep learning model with 3D-Unet (a fully convolutional network in biomedical image segmentation) was used to create preliminary zonal segmentation [18–20], and it was corrected by a radiologist in all cases. Median ADC values of the PZ for each patient were calculated. The median ADC value of the PZ was assumed to represent the ADC value of the normal prostate PZ parenchyma. The variability of the values was used as a surrogate indicator for assessing the influence of technical differences (scanner models and endorectal coil use) on ADC values.

### Statistical analysis

Agreement of ADC values between readers was demonstrated by using Bland-Altman plots with 95% limits of agreement. Difference in 95% limits of agreement among measurement methods were assessed with Friedman test and post hoc tests with Wilcoxon rank sum test adjusted by Bonferroni-Holm method. Inter-observer agreement between measured ADC values by 2D-ROI and computer-calculated reference values was assessed using the intraclass correlation coefficient (ICC). The median ADC values of the PZ and lesions measured under different MRI acquisition conditions were compared by using unpaired t-test. P values less than 0.05 were considered statistically significant. Image and statistical analyses were performed using Python version 3.7 (Python Software Foundation) or R version 3.6.1 (The R Foundation).

## Results

Among 40 lesions, 10 were benign, 6 were GS 6 PCa, and 24 were csPCa. Thirty lesions were in the PZ and 10 were in the TZ. Among 40 lesions, the minimum computer-calculated mean ADC values by different 2D-small-ROIs (mean ± SD [10^−6^ mm^2^/s]) were 635 ± 231 in 1 pixel, 653 ± 226 in 2 pixels, 671 ± 219 in 3 pixels, 693 ± 215 in 5 pixels, 733 ± 186 in 9 pixels, 783 ± 193 in 17 pixels, 809 ± 190 in 25 pixels, 826 ± 192 in 33 pixels and 840 ± 187 in 49 pixels. The computer-calculated 10^th^ percentile ADC value of the 3D-whole-lesion ROI (mean ± SD [10^−6^ mm^2^/s]) was 765 ± 165.

Among the entire cohort of 613 lesions from 466 patients, 378 lesions were benign or GS 6 PCa, 235 were csPCa. 457 were in the PZ and 156 were in the TZ.

**Fig 3:**
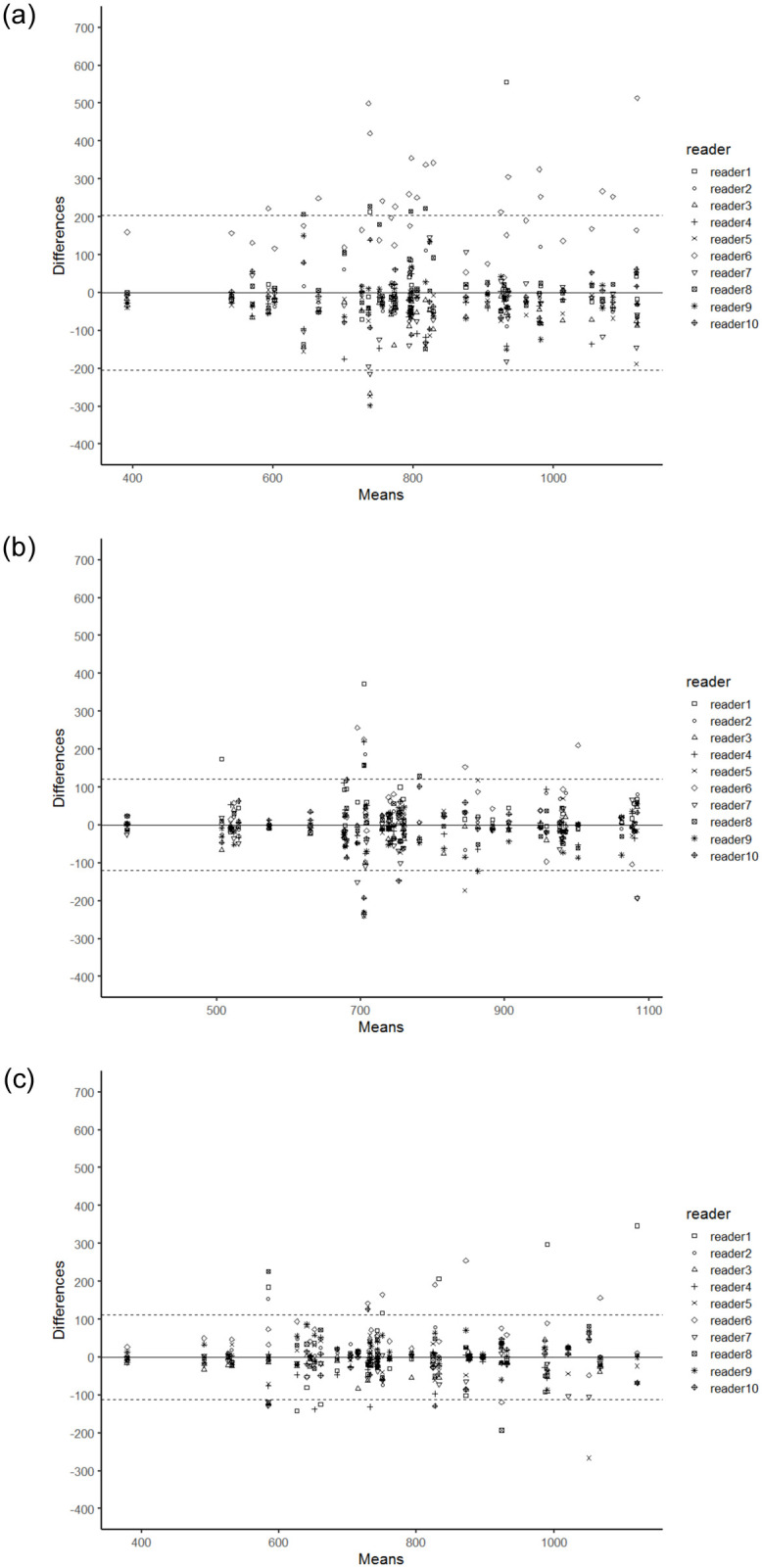
Bland-Altman plots of ADC values in each measurement method. (a), Free 2D-small-ROI method. (b) 2D-small-ROI method with specific instruction to use 9-pixel 2D-small-ROI covering the lowest ADC area. (c) Tenth percentile of 3D-whole-lesion-ROI method. The x-axis is the average ADC value, whereas the y-axis is the difference between ADC value by each reader and the average ADC value. The 95% limits of agreement were ±205 (10^−6^ mm^2^/s) for the free 2D-small-ROI method, ±120 (10^−6^ mm^2^/s) for the 2D-small-ROI method with specific instruction, and ±112 (10^−6^ mm^2^/s) for 10th percentile of 3D-whole-lesion-ROI. ADC indicates apparent diffusion coefficient; ROI, region of interest; 2D, two-dimensional; 3D, 3-dimensional.

The results of the 3 reading sessions are summarized in Table 1. Bland-Altman plots of ADC values in each measurement method are shown in Fig 3. Mean ROI size placed by each reader ranged from 7.5 to 103 mm^2^ in session 1 and 8.0 to 8.9 mm^2^ in session 2. ICC between ADC values measured by each reader and reference computer-calculated values ranged from 0.349–0.919 in session 1 and from 0.736–0.949 in session 2. Mean ADC values across all lesions were 36 to 300 (10^−6^ mm^2^/s) higher than reference computer-based values in session 1 and 25 to 88 (10^−6^ mm^2^/s) higher in session 2. The 95% limits of agreement of ADC values among readers on the Bland-Altman plots were ± 205 (10^−6^ mm^2^/s) for session 1 and ± 120 (10^−6^ mm^2^/s) for session 2. The 95% limits of agreement of ADC values among readers on the Bland-Altman plots were ± 112 (10^−6^ mm^2^/s) for session 3. The difference in 95% limits of agreement of ADC values among 3 measurement methods was statistically significant (P < .001). The post-hoc test demonstrates statistically significant differences between session 1 and session 2 and between session 1 and session 3 (both P < .001), whereas there was no significant difference between session 2 and session 3 (P = .46).

**Table 1:** The results of the multi-reader study.

	**Session 1: Free 2D-small-ROI**		
	ROI size mm^2^ mean±SD (range)	ADC difference from the compuater-calculated reference value (9 pixels) mean±SD	ICC (CI)
**Reader 1**	13.2 ± 9.8 (4.2 to 62.3)	93 ± 142 (-62 to 741)	0.668 (0.316 to 0.836)
**Reader 2**	7.5 ± 6.5 (1.6 to 29.9)	75 ± 97 (-80 to 414)	0.788 (0.398 to 0.910)
**Reader 3**	12.6±18.0 (1.6 to 117.5)	36 ± 64 (-108 to 175)	0.919 (0.800 to 0.963)
**Reader 4**	10.5 ± 6.3 (3.6 to 56.3)	43 ± 82 (-116 to 413)	0.873 (0.719–0.938)
**Reader 5**	8.4 ± 5.1 (1.4 to 24.7)	40 ± 75 (-114 to 185)	0.890 (0.747–0.947)
**Reader 6**	103.0 ± 71.0 (10.0 to 350.0)	300 ± 147 (73 to 717)	0.349 (-0.085 to 0.707)
**Reader 7**	11.9 ± 8.8 (2.4 to 48.5)	44 ± 67 (-67 to 278)	0.909 (0.737 to 0.961)
**Reader 8**	23.3 ± 24.7 (3.2 to 122.4)	118 ± 128 (-84 to 502)	0.614 (0.090 to 0.829)
**Reader 9**	15.8 ± 16.7 (1.8 to 100.0)	68 ± 98 (-78 to 401)	0.808 (0.503 to 0.915)
**Reader 10**	24.2 ± 35.1 (3.3 to 207.0)	74 ± 86 (-71 to 331)	0.818 (0.383 to 0.929)
	**Session 2: 2D-small-ROI with 9 pixel covering lowest ADC value**		
	ROI size mm^2^ mean±SD (range)	ADC difference from the compuater-calculated reference value (9 pixels) mean±SD	ICC (CI)
**Reader 1**	8.2 ± 1.5 (6.0 to 9.8)	88 ± 112 (-80 to 612)	0.736 (0.318 to 0.884)
**Reader 2**	8.2 ± 1.5 (6.0 to 9.9)	72 ± 84 (-61 to 397)	0.839 (0.437 to 0.937)
**Reader 3**	8.2 ± 1.5 (6 to 9.9)	35 ± 50 (-82 to 174)	0.949 (0.824 to 0.979)
**Reader 4**	8.9 ± 1.5 (6.4 to 10.7)	64 ± 86 (-61 to 459)	0.844 (0.539 to 0.934)
**Reader 5**	8.2 ± 1.4 (6.1 to 9.9)	38 ± 56 (-76 to 151)	0.940 (0.810 to 0.975)
**Reader 6**	8.3 ± 1.7 (5.9 to 11.0)	86 ± 115 (-153 to 465)	0.737 (0.345 to 0.882)
**Reader 7**	8.0 ± 1.4 (6.0 to 9.6)	22 ± 66 (-153 to 245)	0.935 (0.875 to 0.966)
**Reader 8**	8.2 ± 1.7 (6.0 to 10.0)	56 ± 83 (-81 to 397)	0.859 (0.615 to 0.938)
**Reader 9**	8.0 ± 1.4 (6.0 to 9.7)	25 ± 63 (-98 to 215)	0.935 (0.868 to 0.967)
**Reader 10**	8.2 ± 1.5 (6.0 to 10.0)	50 ± 67 (-137 to 184)	0.904 (0.675 to 0.961)
	**Session 3: 3D-whole-lesion-ROI (10**^**th**^ **percentile)**		
	ROI volume cm^3^ mean±SD (range)	ADC difference from the compuater-calculated reference value (10^th^ percentile) mean±SD	ICC (CI)
**Reader 1**	0.74 ± 1.29 (0.04 to 7.54)	25 ± 116 (-97 to 166)	0.820 (0.686 to 0.900)
**Reader 2**	0.88 ± 1.61 (0.05 to 7.56)	12 ± 62 (-89 to 256)	0.930 (0.873 to 0.962)
**Reader 3**	0.61 ± 1.05 (0.03 to 5.28)	-15 ± 46 (-97 to 166)	0.960 (0.923 to 0.979)
**Reader 4**	0.70 ± 1.14 (0.03 to 5.61)	-15 ± 53 (-217 to 150)	0.949 (0.905 to 0.973)
**Reader 5**	1.15 ± 1.99 (0.05 to 9.22)	0 ± 58 (-278 to 130)	0.937 (0.884 to 0.966)
**Reader 6**	1.59 ± 1.69 (0.05 to 7.21)	53 ± 90 (-177 to 257)	0.828 (0.608 to 0.918)
**Reader 7**	0.85 ± 1.32 (0.05 to 7.23)	-10 ± 40 (-116 to 73)	0.970 (0.943 to 0.984)
**Reader 8**	2.05 ± 4.70 (0.05 to 27.90)	12 ± 83 (-251 to 329)	0.877 (0.780 to 0.930)
**Reader 9**	1.33 ± 2.12 (0.04 to 10.40)	22 ± 49 (-64 to 167)	0.95 (0.894 to 0.975)
**Reader 10**	0.63 ± -0.93 (0.02 to 5.03)	-4 ± 57 (-97 to 190)	0.946 (0.901 to 0.971)

Abbreviations: ADC: Apparent diffusion coefficient, ROI: Region of interest, ICC: Interclass correlation coefficient, CI: Confidence.

For the entire cohort, median ADC values of the PZ prostate parenchyma were compared under the different MRI acquisition conditions. The scanner models were Discovery MR 750w (GE Healthcare) (n = 275 patients, 349 lesions), Skyra (n = 144 patients, 195 lesions), Discovery MR 750 (GE Healthcare) (n = 38 patients, 59 lesions), Optima MR 450w (GE Healthcare) (n = 2 patients, 2 lesions), Signa HDxt (GE Healthcare) (n = 1 patients, 2 lesions), and unknown model (n = 6 patients, 6 lesions). We used the 2 most commonly used models for comparison (Discovery MR 750w [n = 275] vs Skyra [n = 144]). The means of median ADC values of the 2 models were 1322 vs 1599 (10^−6^ mm^2^/s) (P < .001). In comparison with endorectal coil use (n = 205] vs without endorectal coil use (n = 261), means of median ADC values were 1484 vs 1353 (10^−6^ mm^2^/s) (P < .001). For all lesions, mean computer-calculated ADC reference values using 9-pixel 2D-small-ROI were compared under the different MRI acquisition conditions. For csPCa lesions, mean ADC values were 624 vs 667 (10^−6^ mm^2^/s) (P = .155) for Discovery MR 750w (n = 137) vs Skyra (n = 75). For non-csPCa lesions, mean ADC values were 787 vs 882 (10^−6^ mm^2^/s) (P < .001) for Discovery MR 750w (n = 212) vs Skyra (n = 120).

## Discussion

Our study showed that improvement of interobserver agreement of ADC measurement could be achieved by specifying the 2D-ROI measurement method. In session 2 of the multi-reader study, readers were instructed to use 9-pixel 2D-small-ROI (size specification) covering the lowest ADC area (position specification). The 95% limits of agreement of ADC values among readers were superior with the 2D-small-ROI method with 9 pixels covering the lowest ADC area (± 120 [10^−6^ mm^2^/s]) compared to the free 2D-small-ROI method (± 205 [10^−6^ mm^2^/s]) (P < .001). ICC between ADC values measured by each reader and computer-calculated reference values was also superior with the 2D-small-ROI method with 9 pixels covering the lowest ADC area (0.736–0.949) compared to the free 2D-small-ROI method (0.349–0.919).

In our study, the minimum computer-calculated mean ADC values by 2D-small-ROI were used as representative values to correspond to mean ADC values measured by the 2D-small-ROI that covered the lowest ADC area. The computer-calculated mean ADC values increased as pixel size increased, ranging from 635 (10^−6^ mm^2^/s) in 1 pixel to 840 (10^−6^ mm^2^/s) in 49 pixels. This result suggest that larger 2D-ROI sizes will increase the measured ADC values. PCa is often heterogeneous, and a high-grade component may sparsely present within a low-grade component or non-neoplastic tissue [16,21]. ADC images probably reflect tumor heterogeneity, and using a 2D-ROI to cover the lowest ADC area likely captures the small focus of higher-grade tumors. With larger ROIs, the more lower-grade components would be included, resulting in a higher mean ADC value.

We consider the 2D-small-ROI method with 9 pixels covering the lowest ADC area likely equivalent in interobserver agreement to the 3D-whole-lesion-ROI method with 10^th^ percentile. The 2D-small-ROI method with specific instruction achieved almost the same level of 95% limits of agreement among the readers as the 10^th^ percentile 3D-whole-lesion-ROI method (± 112 [10^−6^ mm^2^/s]) (P = .46). When the 4 best radiologists’ results were used, the 95% limits of agreement among the readers in the 2D-small-ROI method with 9 pixels covering the lowest ADC area were ± 86 (10^−6^ mm^2^/s), slightly better than the 3D-whole-lesion-ROI method (P = .189). The 2D-small-ROI method relies on radiologists’ detection of the small focus of the lowest ADC area. Although it is an intuitive task, careful placement of ROI could probably result in better interobserver agreement. For example, displaying images using a narrow window setting, enlarging images such that they appear pixelated, and placing the ROI on a few different sections may be necessary to find the lowest ADC area. Additionally, placing a small sub-2D-ROI in the lowest ADC area for targeted biopsy could increase the yield of higher grade disease in heterogeneous tumors.

Various factors other than 2D-ROI measurement method could affect ADC values. Previous reports demonstrated that ADC values were inconsistent among different MRI scanner models [13–15,22]. Our study compared median ADC values of the PZ of the prostate parenchyma among different scanner models (Skyra and Discovery MR 750w). ADC values measured on 1 model were lower than the other in all 3 regions; PZ of the prostate parenchyma (277 [10^−6^ mm^2^/s), 27%), csPCa lesions (43 [10^−6^ mm^2^/s), 6%) and non-csPCa lesions (95 [10^−6^ mm^2^/s), 10%). A prior human study compared ADC values of upper abdominal organs between different scanner models, and demonstrated significant differencesin liver, pancreas and kidney, whereas spleen and gallbladder showed no significant difference [22]. Our study suggests that normal prostate gland could also be significantly affected by scanner model. Interestingly, mean ADC values of csPCa lesions with more restricted diffusion were not significant between the 2 models, whereas those of non-csPCa lesions with less restricted diffusion were significant. Similar results were found in a prior phantom study using 6 different scanner models, demonstrating that fluid with less restricted diffusion was affected by scanner model. The difference in ADC values of distilled water was 540 (10^−6^ mm^2^/s) (range: 1885–2425 [10^−6^ mm^2^/s]), which was greater than that of 25% sodium chloride of (314 [10^−6^ mm^2^/s] [range: 1265–1579 {10^−6^ mm^2^/s}]) [13]. Endorectal coil use also affected ADC values in our study, although previous reports showed no significant difference in the ADC of a lesion with or without endorectal coil use [23]. The choice of b values might affect ADC estimates as well [24,25]. Our study did not evaluate ADC values on different b-value sets since we assumed considerable confounding bias in scanner models used in our institution.

In our study, 95% limits of agreement of ADC values among the readers on the Bland-Altman plots were ±205 (10^−6^ mm^2^/s) for the free 2D-small-ROI method, (session 1) and ±120 (10^−6^ mm^2^/s) for the 2D-small-ROI method with 9 pixels covering the lowest ADC area (session 2). Our study was not able to directly compare 95% limits of agreement of ADC values among the scanner models since each lesion was imaged with only 1 scanner. However, we could refer to prior studies investigating ADC difference among scanners in the same area. In the previous phantom study of inter-scanner variability with 4 different fluids scanned by 6 different MRI models by 4 different vendors demonstrated the 95% limits of agreement of ADC values among the scanners were ±306 (10^−6^ mm^2^/s) [13]. In this study, one particular model showed considerably higher values than the other 5 scanners. Among these five scanners, the 95% limits of agreement of ADC values were ±112 (10^−6^ mm^2^/s) [13]. Sasaki et al. reported interscanner variability of ADC values up to 8% in gray and white matter in the same patients with different scanners [14]. Therefore, we can speculate that the influence of interobserver variability on measured ADC value is similar or larger than that of interscanner variability.

Our study had several limitations. First, our study was a retrospective analysis. Second, 3D-whole-lesion-ROI for the computer-generated calculation was retrospectively set by 4 different radiologists, and the border of the lesion varied depending on the radiologists. Third, the number of lesions for the multireader study was relatively small (N = 40). However, 10 radiologists evaluated 40 lesions, so we assume that an appropriate number of data (N = 400) was used for calculating 95% confidence intervals. Moreover, it is assumed that each radiologist had a certain tendency in his or her ADC measurement method, and therefore a similar result would be expected even if the number of evaluated lesions was increased. Fourth, there should exist multivariable confounding factors in the analysis of interscanner difference. The purpose of this analysis was to demonstrate the approximate influence of scanner model difference on ADC values and see how it differed from interobserver variability. Finally, we used arbitrary 9-pixel 2D-small-ROI in this study and the optimal size of 2D-ROI to represent tumor aggressiveness remains an issue.

In conclusion, interobserver agreement of ADC values was superior with the 2D-small-ROI method with 9 pixels covering the lowest ADC area compared to the free 2D-small-ROI method.

## Supporting information

S1 File Annonimized data for the all cohort. (XLSX)

S2 File Annonimized data for the reader study by 10 radiologists for 40 lesions. (XLSX)

## Abbreviations

ADC: apparent diffusion coefficient
csPCa: clinically significant prostate cancer
DWI: diffusion weighted image
FOV: field of view
GS: Gleason score
ICC: intraclass correlation coefficient
PCa: prostate cancer
PI-RADS: Prostate Imaging Reporting and Data System
PZ: peripheral zone
ROI: region of interest
TZ: transitional zone
